# Overcoming navigational challenges: A novel approach to the study and assessment of topographical orientation

**DOI:** 10.3758/s13428-021-01666-7

**Published:** 2021-08-03

**Authors:** Alessia Bonavita, Alice Teghil, Maria Chiara Pesola, Cecilia Guariglia, Fabrizia D’Antonio, Antonella Di Vita, Maddalena Boccia

**Affiliations:** 1 Department of Psychology, “Sapienza” University of Rome, Via dei Marsi, 78, 00185 Rome, Italy; 2 Ph.D. Program in Behavioral Neuroscience, Sapienza University of Rome, Rome, Italy; 3 Cognitive and Motor Rehabilitation and Neuroimaging Unit, IRCCS Santa Lucia, Rome, Italy; 4 Department of Human Neuroscience, Sapienza University of Rome, Rome, Italy

**Keywords:** Spatial navigation, Allocentric, Egocentric, Topographical orientation

## Abstract

Several studies investigating environmental navigation require participants to navigate in virtual environments, in which the proprioceptive and vestibular components present during real environmental navigation are lost. Here, we aimed to provide a novel computerized ecological navigational battery, investigating whether the absence of proprioceptive and vestibular inputs yields a representation of the navigational space comparable to that acquired ecologically. In Study 1, 38 participants underwent two sets of tasks, one performed in a laboratory-based setting (LBS) and the other in an ecological environment (EE), with both including evaluation of route, landmark, and survey knowledge and a landmark ordering task. All tasks, except the route task, significantly correlated between EE and LBS. In LBS, performance in the landmark ordering task was predicted by that in the survey task, but not by those in the route and landmark tasks. Results of Study 1 were replicated in Study 2, in which 44 participants completed a modified and shorter online version of LBS tests. Reliability of the online LBS tests was also tested and showed a moderate-to-high internal consistency. Overall, results show that the conditions in which tasks are performed affect the acquisition of route knowledge, likely due to the lack of proprioceptive and vestibular information in LBS. However, LBS tasks presented here provide a standard battery of tests that can overcome the replicability problems encountered by ecological navigation tests, while taking into consideration all the complexities of navigational processes in terms of the use of landmark, route, and survey strategies.

## Introduction

Spatial navigation is a multimodal process that requires a dynamic integration between perception, attention, memory, and decision-making (Ekstrom et al., 2014). Orienting ourselves within space is essential to maintain the representation of our actual position and our goal destination (Wolbers & Hegarty, 2010). The constant update of spatial positions in time and space is possible thanks to the path integration process, which integrates multiple spatial vectors arising from vestibular and proprioceptive cues, efferent motor commands, and optic and acoustic flow (Angelaki, 2014; Wang & Spelke, 2002). Multiple path integrations that represent several spatial positions are usually combined to form a dynamic representation of objects while we move, thus contributing to the construction of cognitive maps (Wang, 2016). This process underlies the spatial updating system that is pivotal in recognizing objects and locations from a novel point of view during walking (Wang & Spelke, 2002; Wolbers & Hegarty, 2010).

According to the model proposed by Byrne, Becker and Burgess (2007), spatial memory uses egocentric and allocentric information depending on task requirements. A spatial updating system located in the parietal cortex provides egocentric representations of positions and allows one to access long-term memories stored in an allocentric format in the medial temporal areas (Byrne et al., 2007). The vestibular system provides information about head and body movements to this mechanism, collecting such information through receptors in the inner ear that are sensitive to rotational and translational accelerations (Mackrous et al., 2019; McNaughton et al., 2006). Information from vestibular and proprioceptive systems is integrated with information from visual and auditory sensory systems (Chun et al., 2019). In this context, visual inputs are crucial, since they provide a more accurate estimate of the direction in which we are navigating (Cheng & Gu, 2018). The optical flow, namely the set of movement patterns of the image on the retina, gives strong cues about the movement of the body; also, the optical flow can elicit, per se, the illusion of movement (Angelaki, 2014). It is interesting to note that self-generated rotations, such as eye or head movements, alter the images of the environment on the retina; thus, the component of the retinal patterns resulting from these rotations must be eliminated from the visual system to have an optical flow that is informative of the individual's movement and to perceive the world accurately (Sunkara et al., 2015). Visual information can also be used to correct accumulated errors in path integration through the recalibration process (Wang & Spelke, 2002). Although vision alone could guide navigation without the need for any spatial representation, somatosensory and proprioceptive cues are important for computing length and estimating turning angles. Hence, both visual and somatosensory/proprioceptive inputs are necessary to form high-level spatial representations (Ekstrom et al., 2017).

During environmental navigation, besides processing current information from the environment, individuals have to retrieve spatial knowledge from memory. One of the longstanding theories on the acquisition of spatial knowledge posits that it is acquired following three main hierarchical steps, namely *landmark*, *route*, and *survey* representation, with each step incorporating the mechanisms of the previous one (Siegel & White, 1975). Siegel and White (1975) proposed that the internal representations of the environment progress over time from the initial landmark stage to an ultimate stage of survey representation. The landmark representation corresponds to the figurative memory of environmental objects, allowing individuals to navigate towards a salient landmark as a beacon. The route representation includes the mental representation of paths connecting different landmarks in an egocentric perspective. Finally, the survey representation is a map-like representation in which individuals represent spatial relations between landmarks (including Euclidean metrics), regardless of their position. Tversky (1993) proposed the existence of a further stage in which environmental representations are integrated with spatial linguistic categories due to the importance of language for human beings. Montello (1998), instead, criticized the rigid cumulative and hierarchical structure of Siegel and White’s model and the idea that metric knowledge takes so long to develop. Instead, he proposed a “Continuous Model,” in which metric knowledge is continually developed since the first exposure to the environment (Ishikawa & Montello, 2006).

As it appears clearly from the above review of the literature, navigating in a real environment is inherently complex. Indeed, successful navigation involves evaluating our orientation towards the surrounding environment, planning the route beyond the current field of vision, and constantly updating our positions as we move towards the target destination. Thus, it is very difficult to reproduce all of the processes involved in human navigation in controlled laboratory settings (Park et al., 2018). However, evaluating navigational skills in a real environment can also become a challenge. First, it is difficult to create identical conditions for all participants; moreover, potentially interfering factors such as weather conditions, traffic, and noise can be difficult to control. Furthermore, studies testing spatial navigation skills in the real world usually focus on an existing topographic layout, thus assuming the risk not to control for participants’ familiarity with the selected environment (Lopez et al., 2020). Alternatively, a completely new environment can be created; however, this approach is inevitably time-consuming and costly (van der Ham et al., 2015). Assessing navigational skills in a laboratory environment can easily overcome all of these limitations. Research comparing navigation in real and laboratory environments in healthy individuals, in young and elderly groups, and in individuals with traumatic brain injury, cerebral infarction, or dementia (Coutrot et al., 2019), suggests that cognitive processes and brain regions involved in the two types of settings are similar.

In the studies described below, we aimed to provide a novel laboratory-based setting for evaluating spatial navigation using non-immersive virtual environments. With this aim, we investigated whether the mental representations of the navigational space developed in a laboratory-based setting (LBS), in absence of proprioceptive and vestibular inputs, are similar to those acquired ecologically in a real environment (ecological environment, EE). Thus, in the first study, we tested the correlation between performance in LBS and that in EE, and the relation between different mental representations acquired in LBS, to test for possible differences in the way individuals acquire and use spatial knowledge in the two settings. In a second study, we implemented a web-based experiment to replicate previous findings and to provide a feasible LBS to test spatial navigation skills.

## Study 1

### Material and methods

#### Participants

Thirty-eight healthy college students (20 females), aged between 19 and 28 years (*M* = 21.78; *SD* = 2.132), took part in the study. To determine the minimum sample size suitable for this study, we performed an a priori correlation power analysis using G*Power 3.1 (Faul et al., 2009) to achieve a statistical power higher than 95%, considering an alpha of .05. The two-tailed correlation (ρ H1 = −.539) was derived from a previous data set including six healthy subjects (3 females), aged between 24 and 30 years (*M* = 27; *SD* = 2), who followed the LBS protocol used in this study and performed an ecological assessment of spatial navigation skills, namely the Di.Vi.Na. Developmental Topographical Disorientation Battery (DDTD; Bianchini et al., 2010, 2014; Palermo et al., 2014; Boccia, Bonavita, et al., 2019b). The total sample size resulting from the power analysis was *N* = 38. No participants had a history of neurological or psychological disorders, or alcohol or drug abuse. All participants read and signed the informed consent. The study was designed following the principles of the Declaration of Helsinki and was approved by the ethical committee of Fondazione Santa Lucia in Rome.

#### Procedure

The experimental procedure was divided into two sessions with a maximum duration of 120 minutes each. Sessions were held at the Department of Psychology of the Sapienza University of Rome and in the University Hospital "Umberto I" in Rome. To compare computer-based spatial navigation tasks and first-person actual navigation, we used an experimental computerized ecological navigational battery (LBS) and an experimental navigational battery performed in an ecological environment (EE), both of which have proven their efficacy in previous studies (Boccia et al., 2016, 2017; Nemmi et al., 2017; Teghil et al., 2019). The order of administration of EE and LBS was counterbalanced across subjects.

##### Laboratory-based setting

The stimuli used for LBS were taken from an existing city (the city of Latina, near Rome) and were presented to participants on a Windows 7 Dell laptop (16:9 15.60-inch screen, 1366 x 768 resolution, model number: Vostro 3558) using the open-source software “Opensesame” (Mathôt et al., 2012). These materials have already been described extensively in previous studies from our group (Boccia et al., 2015; Boccia et al., 2016; Nemmi et al., 2017; Teghil et al., 2019). None of the participants was familiar with the environment and/or learned the route in real life before the study.

##### Route knowledge

**Fig. 1:**
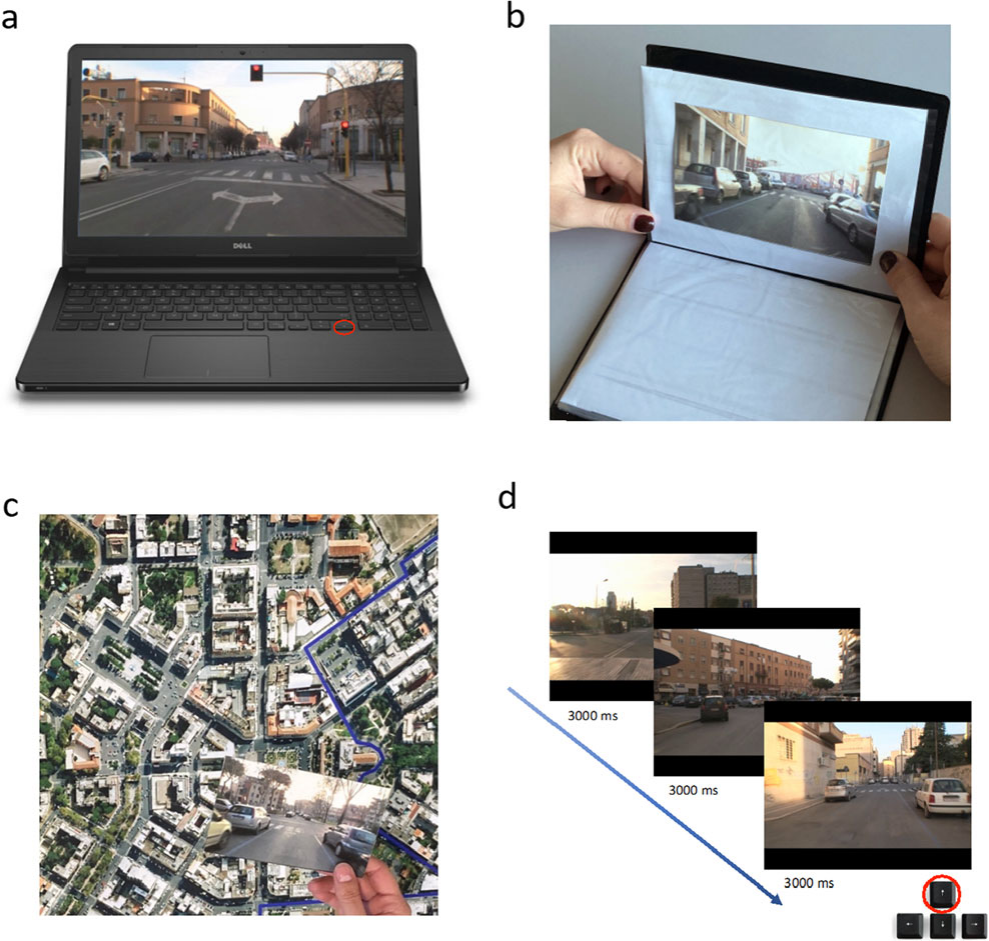
Computerized Ecological Navigational Battery (LBS). **a** Route knowledge task. Participants are shown a video clip of a path filmed in a first-person perspective. At each crossroad, the video stops, and the participant has to choose the correct direction in which to go by pressing the arrow keys on the keyboard. The video starts again only when the participant chooses the correct direction. **b** Landmark knowledge task. Participants have to recognize the crossroads encountered along the path among distractors. **c** Survey knowledge task. A map of the city area in which a printed blue line indicates the path is placed in front of the participant. Participants have to place on the map 23 postcards, derived from the screenshots of the crossroads, in the correct positions along the blue line. **d** Ordering task. The screenshots of the crossroads are presented in an unbroken sequence. Participants are asked to indicate whether the currently displayed crossroad followed or preceded the previous one along the path, pressing one of two keys (forward arrow: after or backward arrow: before)

Participants watched a video filmed by a professional cameraman in Latina. The main feature of the video was that it was shot in a first-person perspective, which gave the viewer the feeling of driving a car through the streets of the city. At each of the 23 crossroads included in the movie, the video stopped, and the participant had to choose the correct direction in which to go (straight, right, or left) by pressing the arrow keys on the keyboard (Fig. 1a). The video started again only when the participant chose the correct direction. The video was presented three times sequentially (Teghil et al., 2019). In the first presentation, participants were told to try to guess the correct answer (first exposure). In the second and third presentations, they were asked to recall the correct directions. A learning score was calculated based on accuracy on the third presentation of the video (Teghil et al., 2019), calculating the sum of correct responses (max = 23). Also, for the reliability analysis, the sum of the correct responses on the second and third attempts was computed (max = 46), similarly to the index used for EE (composite score; see below). These scores represented indexes of route knowledge.

##### Landmark knowledge

During the landmark task, participants had to recognize the crossroads encountered along the path among 46 screenshots (23 representing the crossroads encountered along the path and 23 distractors representing parts of the same city that were not presented in the video) (Fig. 1b). The total number of correct answers (max = 46) was computed as an index of landmark knowledge (Teghil et al., 2019).

##### Survey knowledge

A real map of the city area, created using the satellite vision of Google Maps 2011, was placed in front of the participants. A printed blue line on the map indicated the path. Twenty-three postcards derived from the screenshots of the crossroads were given to participants, who had to put them in the correct positions along the blue line (Fig. 1c). The postcards were presented in random order. No time limit was given, and participants were allowed to change the position of the postcards until they decided they had finished the task. At the end of the session, the experimenter took note of the number of postcards correctly positioned, and in case of errors, he/she showed the correct position of the misplaced postcards. A learning index was calculated based on the total of correct answers given by the participant (max = 23) and used as an index of survey knowledge.

##### Ordering task

Finally, a 1-back task was administered. The screenshots of the crossroads were presented in an unbroken sequence, following a Latin square design, to maximize the combinations of repetitions of the stimuli (Aguirre, 2007; Nonyane & Theobald, 2007). In each trial, stimuli were presented for 3000 ms; based on the learned path, participants had to indicate whether the currently displayed crossroad followed or preceded the previous one along the path by pressing one of two keys (forward arrow: after or backward arrow: before) as quickly and accurately as possible (Fig. 1d). The task included two blocks for a total of 506 trials (242 block 1 and 264 block 2). Every 22 trials, the word “rest” was presented for 3000 ms, allowing the participant to take breaks during the task. As measures of ordering performance, we calculated the sum of correct responses (i.e., accuracy; max = 506) and the mean response time for correct responses.

##### Ecological environment

The experimental procedure was derived from a previous study by our group (Boccia et al., 2017). It included different tasks tapping route, landmark, and survey knowledge of an environment that was novel for the participants. An ordering task was developed as well, mirroring the one described for LBS.

##### Route knowledge

**Fig. 2:**
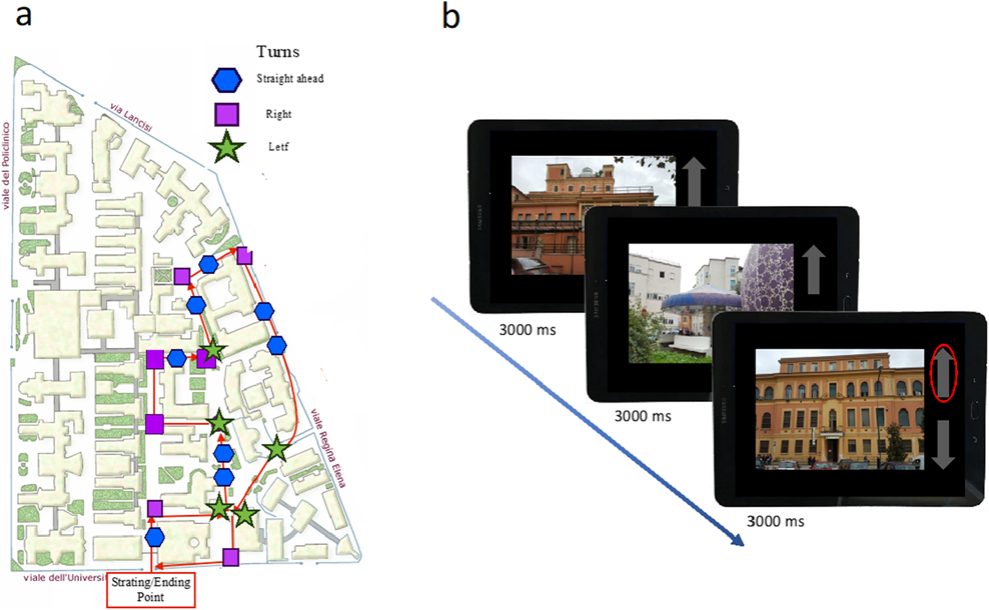
Ecological environment (EE). **a** Map of the path used in the route knowledge task. Participants has to learn a path consisting of 20 turning points balanced across left, right, and straight. The examiner shows the path. When back at the starting point, participants are asked to retrieve it. **b** Ordering task. Photographs of the landmark encountered along the path are presented in an unbroken sequence. Participants are asked to indicate, by pressing one of two keys (forward arrow: after or backward arrow: before), whether the currently displayed landmark followed or preceded the previous one along the path. If the landmark shown is the same as the previous one, they are asked not to press anything

Participants had to learn an outdoor path in the University Hospital shown by the experimenter (Fig. 2a). The path consisted of 20 crossroads with left, right, and straight directions balanced across the task. Before the demonstration, participants were asked to pay attention to each crossroad and landmark on the path. After the demonstration, they were then taken back to the starting point following a different route and asked to retrieve the path. Whenever they took a wrong decision during the retrieval, the experimenter drove them to the last correct turn and asked them to continue. The path was repeated a maximum of three times or until participants demonstrated they had learned it, being able to retrieve the full route without errors (criterion). Similar to Piccardi et al. (2013) for the Walking Corsi Test (WalCT), the learning score (composite score) was calculated by attributing one point for each turn correctly performed until the criterion was reached; then it was added to the score corresponding to correct performance of the remaining trials (up to the third; max: 40). These scores were used as indexes of route knowledge.

##### Landmark knowledge

During the landmark task, we asked participants to recognize the landmarks encountered along the route among 16 photographs (8 representing actual landmarks encountered along the path and 8 representing distractors). As indexes of landmark knowledge, we calculated the total number of correct answers (max = 16) (Boccia et al., 2017).

##### Survey knowledge

Participants were asked to draw the learned route on a blank map. They were provided with a pen and paper map of the University Hospital without any reference. We computed the sum of correct responses (max = 20) as an index of survey knowledge (Boccia et al., 2017).

##### Ordering task

The ordering task was built as a 1-back task, presented on a Samsung Galaxy Tab S3 Android tablet (9.7-inch screen, 2048 × 1536 resolution, model number: SM-T825) using the Android runtime version of “Opensesame” (https://osdoc.cogsci.nl/3.3/manual/android/). The photographs of the landmarks encountered along the path were presented in a randomized and unbroken sequence, following the structure of the 1-back of LBS. In each trial, stimuli were presented for 3000 ms; the task consisted of a total of 64 trials. Based on the learned path, participants were asked to indicate whether the currently displayed landmark followed or preceded the previous one along the learned path by pressing one of two keys (forward arrow: after; backward arrow: before) as quickly and accurately as possible. They were also asked not to press anything if the landmark shown was the same as the previous one (Fig. 2b). Accuracy (i.e., the sum of correct responses, max = 64) was calculated as a measure of ordering performance.

## Data analyses and results

Data were analyzed using IBM SPSS Statistics 25.

### Reliability of LBS

We calculated one-tailed Pearson correlation coefficients between equivalent tasks to investigate the relation between performance in LBS and EE. As specified above, the sum of the correct responses at the second and third attempts for LBS (namely the trials following the first exposure, in which trial-and-error learning was used) and the sum of the correct responses at the first and second attempts for EE were entered in the correlation matrix. The results showed that performance in the route knowledge tasks in EE and LBS was not significantly correlated, *r*(35) = .017; *p* = .461. Instead, performance on the landmark knowledge task in LBS correlated positively with that on the same task in EE, *r*(36) = .511; *p* < 0.001. Also, scores for the survey knowledge task in LBS correlated positively with those for the survey knowledge task in EE, *r*(36) = .454; *p* = .002. Finally, performance in the ordering tasks in LBS and EE was significantly correlated, *r*(36) = .524; *p* < 0.001 (Table 1). The significant association between tasks assessing landmark and survey knowledge and landmark ordering in LBS and EE, confirms the hypothesis that these two conditions test the same processes, providing support for the use of LBS. The absence of vestibular cues in LBS could explain the lack of a significant association between the route knowledge tasks in the two conditions (LBS vs. EE).

**Table 1:** Descriptive statistics and correlation analyses of performance in LBS and EE

Variable	*N*	*M*	*SD*	1	3	5	7
1. Route knowledge EE^a^	38	39.26	1.408				
2. Route knowledge LBS^a^	37	39.03	4.419	.017			
3. Landmark knowledge EE	38	13.13	1.492				
4. Landmark knowledge LBS	38	37.92	4.089		.511**		
5. Survey knowledge EE	38	10.21	8.031				
6. Survey knowledge LBS	38	16.47	6.463			.454**	
7. Ordering EE	38	43.11	10.037				
8. Ordering LBS	38	361.34	66.398				.524**

*Note.* For each task and condition, the number of participants (*N*), the mean (*M*), and the standard deviation (*SD*) of task scores are provided. ^a^Sum of the correct turns on first and second attempts after the first exposure. Significance: * *p* < .05; ** *p* < .01

### Relation between different formats of environmental knowledge

Linear regression analyses assessed the relation between different formats of spatial representation acquired in LBS. Accuracy in the route knowledge task, landmark knowledge task, and survey knowledge task were included as predictors, and accuracy in the ordering task was included as a criterion. Performance in the ordering task was significantly predicted by accuracy in the survey knowledge task (beta = .404; *t* = 2.144; *p* = .039) but not by that in the route knowledge task (beta = .285; *t* = 1.830; *p* = .076) or in the landmark knowledge task (beta = .049; *t* = 0.274; *p* = .786). These results show that only accuracy in the survey knowledge task predicted performance in the landmark ordering task in LBS, a result consistent with that of a previous study using the same paradigm (Teghil et al., 2019), thus providing further support for the reliability of this instrument.

## Study 2

Evaluating navigational ability can become a challenge when testing aged participants, as well as neurological patients presenting multiple cognitive and/or motor impairments. Furthermore, the ongoing COVID-19 pandemic crisis has dramatically highlighted the importance of having tools that can also be used in remote mode and may be easily administered in home settings. With this in mind, this second study aimed to provide an alternative form of usability of LBS, guaranteeing the same efficacy in testing different formats of navigational knowledge.

### Material and methods

#### Participants

Forty-four healthy subjects (23 females) aged between 20 and 32 years (*M* = 24.41; SD = 2.265) took part in Study 2. The sample size was defined a priori using G*Power 3.1 (Faul et al., 2009) to achieve a statistical power higher than 95%, considering an alpha of .05. The effect size (*f*^2^ = 0.784341) was derived from a previous data set (Teghil et al., 2019). The total sample size resulting from the power analysis was *N* = 27. Since the tasks were administered using a web-based platform (see the Procedure section for further details), we enrolled a higher number of participants to deal with possible dropouts (e.g., due to loss of internet connection during the tasks or sudden interruptions). All participants met the same inclusion criteria reported for Study 1. The study was designed following the principles of the Declaration of Helsinki and was approved by the ethical committee of Fondazione Santa Lucia in Rome. All participants provided digital informed consent.

#### Procedure

The experimental procedure consisted of a modified and shortened version of LBS tasks described above. Due to the ongoing pandemic crisis, all the stimuli were presented using the online experiment builder “Testable” (https://www.testable.org/). The stimuli used and the .csv file of the paradigm used in this study are available at https://osf.io/8xt2e/?view_only=6319f4a37d2f4cd996ce889b32a2aa6f. The original paradigm was reduced in length and only 9 out of the original 23 crossroads were used (namely those from crossroad number 7 through crossroad number 15). The selected crossroads were balanced for the number of turns (left, right, or straight) with respect to the original version. To verify whether the same pattern of relations between navigational tasks observed in the original paradigm was maintained in this shortened version, we re-analyzed data from our previous study in which the LBS paradigm was used (Teghil et al., 2019) and performed a correlation analysis between performance in the different tasks, only considering performance for the path selected for the shortened version (crossroads 7–15). Results showed no significant correlation between performance in the route knowledge task and in the other tasks (accuracy in the landmark knowledge task: *r*(25) = .012; *p* = .952; accuracy in the survey knowledge task: *r*(25) = −.221; *p* = .269; accuracy in the landmark ordering task: *r*(22) = −.366; *p* = .078; response time in the landmark ordering task: *r*(22) = .114; *p* = .597). A significant correlation was found between accuracy in the landmark knowledge task and the landmark ordering task response time, *r*(22) = −.432; *p* = .035, but not between accuracy in the landmark knowledge task and that in the other tasks (accuracy in the survey knowledge task: *r*(25) = .317; *p* = .107; accuracy in the landmark ordering task: *r*(22) = .199; *p* = .351). A significant correlation was also found between accuracy in the survey knowledge task and accuracy in the landmark ordering task, *r*(22) = .473; *p* = .019, but not response time in the landmark ordering task *r*(22) = .185; *p* = .387. These results are consistent with the original pattern (Teghil et al., 2019). Since the paradigm has been slightly modified, only the tasks most affected by these modifications will be described in detail below. As for Study 1, accuracy was calculated using the sum of correct responses in each task.

##### Survey knowledge

**Fig. 3:**
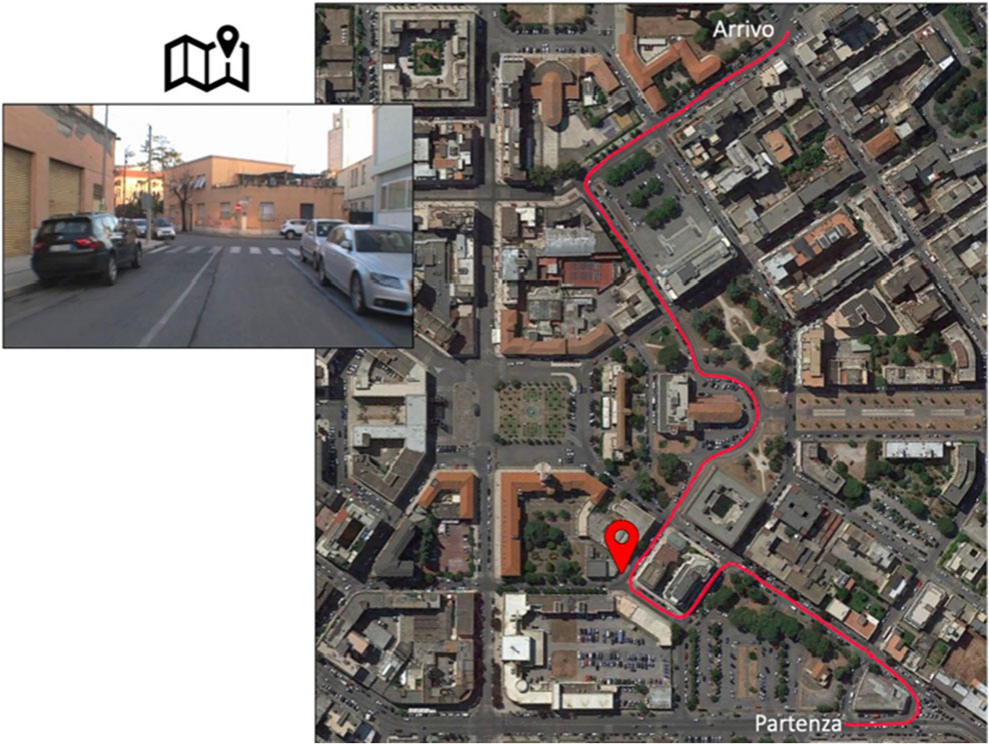
Modified survey knowledge task. The figure shows an example of the stimuli presented to the participant in the survey knowledge used in Study 2. The participant has to indicate whether the placeholder on the map corresponds to the actual position of the crossroad shown in the upper left corner

Each trial of this new computerized version presented (1) a screenshot depicting one of the crossroads encountered along the path and (2) the real map of the city area surrounding the path. The path was indicated on the map using a red line, and a placeholder was shown corresponding to the position of one of the crossroads (Fig. 3). Participants had to indicate whether the position of the presented crossroad corresponded to that occupied on the map by the placeholder, by pressing the 'S' key to say yes or the 'N' key to say no. The sum of the correct answers was computed and used as an index of survey knowledge (max = 18).

##### Ordering task

The 1-back task was also modified. Following the original task structure, the screenshots of the crossroads were presented in a randomized and unbroken sequence (Aguirre, 2007). In each trial, stimuli were presented for 3000 ms for a total of 81 trials divided into three blocks (first block = 27; second block = 27; third block = 27). Based on the learned path, participants were asked to indicate whether the currently displayed crossroads followed or preceded the previous one, by pressing one of two keys (forward arrow: after or backward arrow: before) as quickly and accurately as possible (experimental trials); they were asked to press the space bar if the crossroad was the same as the previous one (*N* = 6) or if it was the first of the block (*N* = 3). Accuracy was computed as the sum of correct responses (max = 72) on experimental trials and used as an index of ordering performance.

## Data analyses and results

Data were analyzed using IBM SPSS Statistics version 25. Mean, standard deviation, and the number of participants for each task are reported in Table 2.

**Table 2:** Spatial navigation assessment, shortened version

Task	*N*	Max	Accuracy	
			*M*	*SD*
Route knowledge	44	9	7.91	1.64
Landmark knowledge	44	18	16.48	1.798
Survey knowledge	44	18	14.36	3.066
Ordering	44	72	46.95	16.944

*Note.* For each task, the number of participants (*N*), the maximum score for each task (*max*), the mean (*M*), and the standard deviation (*SD*) are reported

### Reliability of the shortened LBS

First, we verified the internal consistency for the task adaptation. The route knowledge task showed a good internal consistency (α = .737), as did the survey knowledge task (α = .753); the landmark knowledge task showed a moderate internal consistency (α = .657), whereas the ordering task showed a high internal consistency (α = .957). These results are consistent with those of Study 1; furthermore, they confirm what was found in a previous study (Teghil et al., 2019), suggesting that the modified battery of tasks maintains its reliability also in this shortened version, showing an overall good internal consistency.

### Relation between different formats of environmental knowledge

A linear regression was performed, with the performance in the landmark ordering task as the criterion and the performance in route knowledge, landmark knowledge, and the survey knowledge tasks as predictors. Results showed that accuracy in the landmark ordering task was significantly predicted by accuracy in the survey knowledge task (beta = .489; *t* = 2.643; *p* = .012) but not by that in the route knowledge task (beta = −.031; *t* = −0.172; *p* = .864) or in the landmark knowledge task (beta = .158; *t* = 1.106; *p* = .275). Thus, accuracy in the landmark ordering task was again predicted only by that in the survey knowledge task.

## General discussion

The present studies aimed to provide a new LBS for the assessment of navigational skills. With this goal in mind, the first step was to test the relation between tasks in LBS and similar tasks in EE to verify whether in LBS, the absence of proprioceptive and vestibular inputs negatively affects the acquisition and recall of the mental representations of the navigational space.

Most of the investigated representations in LBS, namely the landmark and survey knowledge, were found to be correlated with their counterparts in EE. Also, performance in the landmark ordering task in LBS and EE was highly correlated. However, we found that the level of route knowledge measured in LBS did not correlate with that measured in EE. This is likely due to the fact that processing of vestibular cues, which is fully available in EE, plays an important role in acquiring route knowledge. Providing analogous vestibular inputs is not possible in LBS; thus, this suggests that using an LBS condition may allow us to fully evaluate navigational skills but not to assess the role played by the so-called idiothetic information processing (the processing of proprioceptive and vestibular inputs). Thus, LBS must be used with caution in evaluating some of the navigational processes—namely the path integration process, in which the processing of vestibular-proprioceptive cues about individual movements in the environment is essential—but nonetheless provides a very reliable way to test re-orienting, landmark, and survey/allocentric processing.

Concerning the relation between investigated representations (i.e., landmark, route, and survey knowledge) and the landmark ordering task, our results are consistent with previous studies that used similar materials and procedures (Boccia et al., 2017; Teghil et al., 2019), suggesting that both navigational assessment conditions, namely LBS and EE, test the same process. This is consistent with studies reporting that the spatial information presented in a virtual environment is sufficient to generate the same spatial representation acquired in an ecological environment (Arnold et al., 2013; Coutrot et al., 2019).

Similarly to Teghil et al. (2019), LBS accuracy in the ordering task was not significantly predicted by either landmark knowledge or route knowledge, demonstrating that the ordering task does not depend upon these representations. Instead, the survey knowledge significantly predicted accuracy in the landmark ordering task, demonstrating that this type of mental representation of the environment has a closer relation with the ordering task. Finding that landmark ordering in LBS was significantly predicted only by survey knowledge—although information about the order of landmarks is usually assumed to be part of route knowledge (Siegel & White, 1975; Montello, 1998; van Asselen et al., 2006; Nemmi et al., 2013)—likely reflects the reliance on an abstract representation of the path, possibly due to the lack of vestibular, kinesthetic, or motion cues during route learning (Teghil et al., 2019). Notably, this result emerged from both the original and shortened versions of LBS.

During navigation, landmarks are separated from other relevant objects in the environment and associated with decision points along the path where a direction must be taken; therefore, a landmark knowledge task evaluates the ability to recognize landmarks having salient visual and geometric features that allow an individual to distinguish them from their surroundings (Arnold et al., 2013), as well as a strong locational/directional connotation (Farrell, 1996); this ability could be particularly relevant in real environments, in which locomotion allows a more exhaustive inspection of the environmental layout. However, it is important to point out that a spatial representation of the path can also be fostered by tasks performed in a laboratory environment. Path integration can also be obtained by relying only on the optical flow, which provides sufficient information for the formation of a representation of the path (Arnold et al., 2013; Harris & Wolbers, 2012; Kearns et al., 2002) and can elicit strong visually-induced illusions of self-motion (Brandt et al., 1973). Moreover, functional magnetic resonance imaging (fMRI) studies show that brain regions sensitive to optical flow increase their interaction with brain regions critical for spatial navigation when participants perform a goal-directed navigational task in a first-person perspective (Sherrill et al., 2015). Also, the strength of this communication increases during the visual path integration process based on self-perceived spatial navigation ability (Zajac et al., 2019). In the learning task we developed for LBS, participants experienced videoclips from a first-person perspective; therefore the optical flow was guaranteed.

Of note, topographical disorientation may occur as a consequence of brain damage, often in patients also showing motor impairments (Bernspang et al., 1987; Bocchi et al., 2020; De Nigris et al., 2013; Grigoryeva & Tikhomirov, 2019; Vuilleumier, 2007). Recent studies on spatial cognition in subjects with cognitive disorders show the benefits of using virtual reality to assess navigation strategies (Buxbaum et al., 2008, 2012; Cogné et al., 2017). However, patients who have suffered from brain damage (e.g., traumatic brain injury or stroke patients) or who are affected by cognitive impairments (e.g., Alzheimer’s disease or mild cognitive impairment) often have problems in different cognitive domains and sensory-motor impairments, which prevent the assessment in a real ecological environment. Thus, the laboratory-based battery we propose here could be a useful tool to evaluate navigational abilities in patients unable to move, and in all those cases in which a test in an ecological environment would be impossible; this could be especially important to improve early diagnosis of neurodegenerative disorders known to affect spatial navigation skills, such as mild cognitive impairment (Boccia, Di Vita, et al., 2019a). In particular, the short version of LBS developed in Study 2 could thus be an excellent tool to assess navigational skills in such patients. The data analysis shows complementarity with what was found in Study 1 and with previous studies (Teghil et al., 2019), confirming the reliability of the tool used, guaranteeing savings in terms of administration time, and increasing the feasibility and usability of the tool.

Also, considering the complementarity between allocentric and egocentric spatial representations and how navigation strategies can be selectively influenced according to the neural networks that have been damaged (Doeller et al., 2008; Ekstrom et al., 2014; Gomez et al., 2014), evaluating different navigational representations (i.e., landmark, or route and survey formats) is mandatory. In this respect, the instrument we propose in the present paper provides a comprehensive investigation of the different formats of spatial representation, also allowing us to test for possible dissociations between them.

It is worth noting that, although performance in LBS presented here was found to be highly correlated with that in EE, the contribution provided by vestibular and proprioceptive cues in actual route navigation cannot be reproduced in this kind of laboratory setting. To overcome this limitation, an exhaustive evaluation of navigational skills, combining the present LBS with other tests, would be useful and would allow us to assess the learning of sequences of positions in space (e.g., the WalCT, Piccardi et al., 2008). We also have to consider that there are some differences between EE and LBS that could further explain the lack of correlation between the route tasks in the two settings. In the LBS route task, participants have a forced visual field, since they are watching videoclips. On the one hand, this inevitably creates a difference with actual navigation, in which the visual field is not fixed. On the other hand, this feature has the important advantage of allowing us to exert stricter control on potentially confounding variables, which is much more difficult in ecological environments, where participants don't have limitations in the visual field and can freely look at any beacon along the way. Finally, another difference between EE and LBS route tasks is response modality. Whereas in the first presentation of the EE route task participants followed the experimenter along the route, responses to the first presentation of the video in LBS are given following a trial and error procedure. We included such a condition to have a control over the task and to monitor the participant's activity. Also, it includes an active task to be performed by participants, which ensures that they actually process the correct direction (i.e., the video started again only when the participant chose the correct direction).

## Conclusions and future directions

The results of the present studies offer new perspectives on the experimental and clinical assessments of spatial navigation abilities. The LBS battery we provide here allows for evaluation of landmark, route, and survey strategies presenting a real environment in a virtual way. At variance with most of the virtual environments described in the literature, the real environment we use in LBS provides a complex, rich stimulus that mirrors the experience individuals live when visiting a novel city. Its portable setting allows testing of participants with a standard battery even when they cannot reach the evaluation site; also, it overcomes possible difficulties in building up paths of equal difficulty for individuals coming from different places. To date, all studies testing topographical disorientation used ad hoc built paths within the cities where individuals lived (see for example Bianchini et al., 2010; Bianchini et al., 2014; Caffò et al., 2020; Conson et al., 2018; Iaria et al., 2009; Palermo et al., 2014), or virtual towns (e.g., Barclay et al., 2016; Burles & Iaria, 2020; Iaria et al., 2014; Iaria & Barton, 2010). It has been pointed out that carrying out a test in an ecological environment in the same city in which participants live can be contraindicated since it can be time-consuming and, above all, makes it difficult to control for familiarity with the environment, a factor that adds noise to the measurements (van der Ham et al., 2015).

The instrument described here overcomes these limitations. Overall, the LBS we described above provides a standard test that can overcome the replicability problems encountered by ecological navigation tests while taking into consideration all the complexities of navigational processes in terms of the use of landmark, route, and survey strategies, as well as the difficulty of navigating in a sophisticated real environment. A standardization of LBS in different age groups is thus warranted for use in future studies to improve its application also in clinical settings.

## Data Availability

Materials for the experiment of Study 2 are available at https://osf.io/8xt2e/?view_only=6319f4a37d2f4cd996ce889b32a2aa6f. Data for all experiments are available from the corresponding author upon request. None of the experiments was preregistered.
